# Syndrome of Inappropriate Antidiuretic Hormone (SIADH) in Chronic Respiratory Diseases: A Comprehensive Review

**DOI:** 10.7759/cureus.77407

**Published:** 2025-01-13

**Authors:** Khaled Al Zaman, Aaesha A Alhebsi, Abdulaziz Almheiri, Hind Alhosani, Nada T Alshehhi, Eissa S Alwheibi

**Affiliations:** 1 Internal Medicine, Cleveland Clinic Abu Dhabi, Abu Dhabi, ARE; 2 Internal Medicine, Cleveland Clinic Abu Dhabi‎, Abu Dhabi, ARE; 3 Medicine, Cleveland Clinic Abu Dhabi‎, Abu Dhabi, ARE; 4 Surgery, Cleveland Clinic Abu Dhabi‎, Abu Dhabi, ARE

**Keywords:** chronic obstructive pulmonary disease, chronic respiratory diseases, hyponatremia, siadh, syndrome of inappropriate antidiuretic hormone secretion (siadh), tuberculosis

## Abstract

The syndrome of inappropriate antidiuretic hormone secretion (SIADH) is a complex and often underdiagnosed disorder characterized by impaired water homeostasis, leading to hyponatremia and associated complications. This comprehensive review explores the intersection of SIADH with chronic respiratory diseases, including chronic obstructive pulmonary disease (COPD), pulmonary tuberculosis, cystic fibrosis, and interstitial lung disease. The review looks at current evidence on pathophysiology, diagnostic challenges, and treatment approaches, highlighting the need for specialized management strategies to improve patient outcomes. Through an analysis of clinical and observational studies, this review highlights the significant impact of SIADH on morbidity and mortality among patients with respiratory diseases. It illustrates the necessity for further research to refine diagnostic and therapeutic modalities.

## Introduction and background

The syndrome of inappropriate antidiuretic hormone secretion (SIADH) may be a clinical disorder characterized by excessive antidiuretic hormone (ADH) secretion, leading to water retention, dilutional hyponatremia, and electrolyte imbalances. SIADH has long been related to conditions such as malignancies, central nervous system disorders, and certain drugs, but its event in chronic respiratory diseases has picked up expanding consideration. Chronic respiratory conditions such as chronic obstructive pulmonary disease (COPD), interstitial lung disease (ILD), and pulmonary tuberculosis (PTB) are related to systemic irritation, hypoxia, and modified pulmonary function, all of which may contribute to the advancement of SIADH. Understanding the association between these respiratory illnesses and SIADH is vital for progressing clinical management and understanding outcomes [1].

COPD, characterized by persistent airflow restriction and constant irritation, is habitually complicated by hypoxia and systemic irritation. These components can disturb the hypothalamic-pituitary axis, which controls ADH secretion, leading to improper release of ADH. Essentially, ILD, which includes dynamic scarring of lung tissue, can result in hypoxemia, lung diseases, and systemic inflammation factors that will moreover trigger SIADH. PTB is a persistent infectious disease that can lead to systemic inflammation and changes in fluid balance, potentially speeding up the development of SIADH. Hence, unremitting respiratory diseases are progressively recognized as contributing variables to SIADH, although the full extent of this relationship remains underexplored [2].

The pathophysiology of SIADH in persistent respiratory illnesses is multifactorial. Hypoxia may be a common feature in many chronic lung diseases and could be a noteworthy trigger for ADH release. Hypoxia activates the sympathetic nervous system and inflammatory pathways, which can stimulate the posterior pituitary gland to release ADH improperly. Furthermore, cytokines and other inflammatory mediators released during lung infection can impact the central nervous system and worsen ADH emission. In a few cases, impeded renal work, frequently seen in patients with persistent lung illness, may also prevent the kidneys' capacity to excrete water, compounding SIADH's fluid retention and hyponatremia characteristics [3].

Clinically, SIADH in patients with persistent respiratory illnesses frequently presents with symptoms such as nausea, headache, confusion, seizures, and, in severe cases, coma. The diagnosis of SIADH can be challenging, especially in patients with persistent respiratory conditions who may already have symptoms such as fatigue or confusion. Key diagnostic criteria incorporate hyponatremia with low plasma osmolality, improperly concentrated urine, and the absence of other causes of water retention, such as dehydration or renal failure. It is fundamental to consider SIADH within the differential conclusion when managing patients with unexplained hyponatremia, especially those with chronic lung illness [4].

The management of SIADH in patients with persistent respiratory illnesses includes addressing the underlying electrolyte disturbances and the respiratory condition. The goal is to correct hyponatremia and prevent complications such as neurological disability. Treatment regularly incorporates fluid restriction, administration of hypertonic saline in extreme cases, and the use of vasopressin receptor antagonists (vaptans) to block the impacts of ADH. However, individualization is necessary to avoid osmotic demyelination, a major consequence caused by rapidly correcting sodium levels. Viable management also includes controlling the underlying respiratory illness, as exacerbations can exacerbate SIADH symptoms and increase the chance of complications [5].

This comprehensive review provides a detailed analysis of SIADH in the setting of persistent respiratory illnesses, centering on its pathophysiology, diagnosis, and management. With the increasing recognition of SIADH in persistent lung illness patients, healthcare professionals must be vigilant in diagnosing and managing this disorder to improve patient outcomes. Understanding the relationship between chronic respiratory illnesses and SIADH will improve clinical decision-making and ultimately benefit patients who are affected.

## Review

Methodology

Research Question and Search Strategy

This study aims to address the following research question: What is the relationship between SIADH and chronic respiratory diseases, and how do its pathophysiological mechanisms, clinical presentations, diagnostic challenges, and management strategies interact in affected patients?

A comprehensive literature search was conducted across multiple databases, including PubMed, Scopus, and Web of Science, to identify relevant studies published between January 2000 and December 2024. Keywords including “SIADH”, “Syndrome of Inappropriate Antidiuretic Hormone Secretion”, “hyponatremia”, “chronic respiratory diseases”, “COPD”, “pulmonary tuberculosis”, “cystic fibrosis”, “pneumonia”, and “interstitial lung disease” were used to construct the search query.

Search Query

The search terms were as follows: ( TITLE-ABS-KEY ( siad OR siadh OR "syndrome of inappropriate antidiuretic hormone secretion" ) AND TITLE-ABS-KEY ( "hyponatremia" OR "respiratory" OR "chronic respiratory disease*" OR "COPD" OR "pulmonary tuberculosis" OR "cystic fibrosis" OR "pneumonia" OR "interstitial lung disease" ) AND NOT TITLE-ABS-KEY ( "kidney" OR "renal" OR "heart" OR "cardiology" OR "endocrine" OR "case report" OR "children" ) ) AND ( LIMIT-TO ( SRCTYPE , "j" ) ) AND ( LIMIT-TO ( PUBSTAGE , "final" ) ) AND ( LIMIT-TO ( SUBJAREA , "MEDI" ) ) AND ( LIMIT-TO ( DOCTYPE , "ar" ) OR LIMIT-TO ( DOCTYPE , "re" ) ) AND ( LIMIT-TO ( LANGUAGE , "English" ) ).

Study Selection

An initial search on the Scopus database identified 406 studies. The authors evaluated the studies for relevancy and applicability to the research question. This resulted in the exclusion of 336 studies, leaving 70 articles to be selected for the review article.

After retrieving the initial search results, duplicate records were removed using reference management software. Two reviewers independently screened titles and abstracts to identify articles that addressed the research question. This was followed by a detailed review of the full-text articles to assess their eligibility based on the inclusion and exclusion criteria.

Studies were included if they focused on the relationship between SIADH and chronic respiratory diseases and provided insights into pathophysiological mechanisms or clinical management. The inclusion process also considered studies addressing outcomes such as morbidity, mortality, and quality of life in patients with SIADH linked to respiratory diseases. Only peer-reviewed English-language papers were considered, focusing on clinical trials, cohort studies, case-control studies, and systematic reviews that provided empirical data or comprehensive analysis. Sources such as preprints, book chapters, and conference abstracts were excluded. Articles were excluded if they lacked sufficient data or focused on unrelated etiologies of SIADH, such as malignancies or drug-induced cases. Furthermore, articles focusing on pediatric cases were excluded to maintain the review's focus on adult populations.

Data Charting and Analysis

Data from the selected studies were systematically extracted into key categories, including study characteristics, pathophysiological mechanisms, clinical features, management strategies, and outcomes. A narrative synthesis was conducted to identify common patterns and themes across the studies, integrating findings into a coherent analysis.

Clinical presentation of SIADH in respiratory diseases

SIADH has a non-specific clinical presentation, making early recognition challenging. It is usually associated with hyponatremia, leading to cerebral edema. Nausea, malaise, and vomiting are among the earliest signs of hyponatremia, usually present when sodium levels are between 125 and 130 mEq/L. When sodium concentration drops suddenly, symptoms of drowsiness, lethargy, headache, and seizures occur. On the other hand, chronic hyponatremia can be asymptomatic due to cerebral adaptation, which could mask hyponatremia levels even below 120mmol/L. Assessing skin turgor, mucus membrane dryness, and the presence of leg edema can help differentiate SIADH from hypovolemic and hypervolemic hyponatremia. Moreover, checking random blood glucose levels can help rule out pseudo-hyponatremia. Moreover, patients with SIADH are usually euvolemic, making their diagnosis subtle. The diagnosis of SIADH can be guided by the Schwartz and Bartter clinical criterion, established in 1967 (Table 1), and still regarded as relevant today [6-10].

**Table 1:** The Schwartz and Bartter clinical criterion ADH, antidiuretic hormone

Criterion	Description
Serum sodium	Less than 135 mEq/L
Serum osmolality	Less than 275 mOsm/kg
Urine sodium	Greater than 20-40 mEq/L (due to ADH-mediated free water absorption from renal collecting tubules)
Urine osmolality	Greater than 100 mOsm/kg
Clinical evidence of volume depletion	Absence of clinical signs: normal skin turgor and blood pressure within the reference range
Other causes of hyponatremia	Absence of adrenal insufficiency, hypothyroidism, cardiac failure, pituitary insufficiency, renal diseases with salt wastage, hepatic diseases, or drugs impairing renal function

A study published by the Journal of Nephrology showed that clinical assessment alone has low sensitivity (50-80%) and specificity (30-50%) [11]. For this reason, combining all the findings will result in the highest accuracy when classifying and treating hyponatremia. Moreover, fraction uric acid excretion (FeUe) and the challenge of volume expansion are two novel diagnostic tests that can differentiate between the three classifications of hypotonic hyponatremia.

In patients with lung disorders, diagnosis can be even more challenging due to hypoxemia, which can present with increased breathing work, drowsiness, and lethargy. However, neurological manifestations such as nausea, vomiting, and seizures are not related to hypoxemia and hypercapnia, which alert physicians to manage SIADH immediately [10].

SIADH in asthma and COPD

Pulmonary diseases can lead to SIADH, although the mechanism by which this occurs is not clear. A similar response may infrequently be seen with asthma [12]. ADH may be elevated in acute asthma, leading to significant hypo-osmolality and hyponatremia, with adverse effects on the patient [13]. Factors that might have contributed to the onset of SIADH include hypoxemia, pulmonary infection, hemodynamic abnormalities, and possibly pharmacological agents and stress [12]. The clinical manifestations of SIADH are unspecific and associated with bodily fluids' hypo-osmolality. The more symptoms a patient has, the faster this ailment progresses. Confusion, obtundation, and seizures are nearly always linked to serum sodium levels below 115 mmol/L. Symptoms of moderate hyponatremia can include overall malaise, appetite loss, weariness, and some degree of cognitive impairment [14].

COPD is a chronic inflammatory illness in which extrapulmonary symptoms might affect the prognosis. Hospitalization for a COPD exacerbation is frequently accompanied by hyponatremia, which is linked to a worse clinical outcome. COPD is one of the diseases that might be associated with SIADH. Although hypoxia has been associated with ADH secretion, hypercapnia is more frequently associated with this condition [15].

SIADH in tuberculosis

Patients with PTB may develop SIADH, and previous studies have shown an association between hyponatremia and increased mortality risk in patients with PTB. A major concern in SIADH is that the administration of isotonic saline infusion may worsen hypotonic hyponatremia via the "desalination" phenomenon. Hypertonic saline is preferred, and the patient's fluid intake should be limited. Therefore, understanding the frequency and risk factors for hyponatremia in patients with PTB is crucial for improving their clinical outcomes [16]. A prospective observational study was conducted on patients with PTB. A total of 100 patients were enrolled in the study who met the predefined inclusion criteria of age more than 18 years and having been diagnosed with active tuberculosis. According to the study's findings, 4% of people had SIADH and 44% had hyponatremia. Although mild hyponatremia was seen in more than 50% of patients, it was found that hyponatremia in PTB was detected in 44% of the patients, with male preponderance. The predominant mechanism of hyponatremia was SIADH, which was present in 65% of cases with hyponatremia [17]. In 1969, a study demonstrated that 11% of patients with active tuberculosis had hyponatremia, with SIADH being the primary cause. Lee et al. described an uncommon instance of PTB presenting as hyponatremia, with biochemical evidence suggesting ectopic ADH synthesis as a probable cause. Detecting and treating electrolyte abnormalities in individuals with respiratory symptoms is critical. When dealing with electrolyte abnormalities in patients who exhibit these symptoms, healthcare providers should include tuberculosis in the differential diagnosis [18].

SIADH in cystic fibrosis

Cystic fibrosis (CF) is an autosomal recessive disorder caused by loss-of-function mutations in the CF transmembrane conductance regulator (CFTR) gene, which encodes a chloride and bicarbonate channel [19]. The syndrome of inappropriate secretion of ADH was observed in patients with CF during acute exacerbation of chronic pulmonary disease; however, it may be underdiagnosed [20,21].

A case report of a 25-year-old male with CF presenting with an acute pulmonary exacerbation demonstrated features of SIADH. His respiratory insufficiency necessitated therapy with intravenous (IV) antibiotics and bi-level, non-invasive, positive-pressure ventilation during his several extended hospital stays. On admission, his serum sodium was 123 mmol/L with serum chloride 74 mmol/L, total osmolality 256 mmol/kg, and urine osmolality 425 mmol/kg. His recent sputum culture results demonstrated *Pseudomonas aeruginosa*. This inappropriately high urine osmolality in the presence of hyponatremia was taken to confirm the diagnosis of SIADH. The treatment from day 2 was the cessation of IV fluids and restriction of oral fluids to 1 L per day. A small response in serum sodium and urinary osmolality was seen, with serum sodium increasing to 124 mmol/L and urinary osmolality falling to 377 mmol/kg. For the management of SIADH, oral fluid was restricted to 750 mL daily, including free water restriction. Serum sodium gradually normalized, returning to 135 mmol/L. This instance of hypovolemic hypoelectrolytemia with SIADH in the context of an abrupt respiratory exacerbation of CF emphasizes the significance of evaluating another diagnosis for hyponatremia. Low serum sodium in CF might result from electrolyte depletion or a dilutional impact from free water retention due to SIADH. In this case, positive-pressure ventilation and bacterial lung infection coexisted, both known to induce SIADH. Acute CF exacerbations can cause dehydration, hyponatremia, and hypochloremia. Diagnosing SIADH has therapeutic implications since it requires fluid restriction rather than fluid resuscitation [21].

SIADH in pneumonia

Pulmonary infections such as community-acquired pneumonia (CAP) are one of the most common causes of hyponatremia. Infectious entities include viral or bacterial pneumonia, tuberculosis, and pulmonary abscesses. Common bacterial pathogens include *Legionella pneumophila* and *Streptococcus pneumoniae*, whereas viral pathogens include respiratory syncytial virus, rhinovirus, adenovirus, influenza virus, and more recently SARS-CoV-2 (COVID-19) [22,23].

Historically, the mechanism by which lung disorders such as pneumonia cause SIADH is unknown. However, recent studies suggest that the pathogenesis for the development of SIADH in SARS-CoV-2 pneumonia can be due to the production of proinflammatory cytokines such as interleukin-6, which doubles the production of ADH via direct stimulation of non-osmotic secretion of ADH, and the activation of hypoxic pulmonary vasoconstriction pathway due to direct insult to the alveolar basement membrane. Further studies are needed to confirm this mechanism [23].

The management of pneumonia-induced SIADH includes fluid restriction and supportive management, in addition to the appropriate treatment of pneumonia, whether viral or bacterial.

SIADH in lung cancer

The association between lung malignancy and SIADH was first reported in 1957. SIADH is a known paraneoplastic syndrome that occurs with small cell lung carcinoma (SCLC). Multiple studies reported that the incidence rate of SIADH in patients with SCLC is 10-15%. Furthermore, it is associated with poorer prognosis in patients with lung cancer who are hospitalized and those with advanced disease [1].

Ectopic secretion of ADH by malignant cells is the most common mechanism for the development of SIADH. Furthermore, SIADH is not only confined to paraneoplastic syndrome caused by SCLC; the use of chemotherapy agents such as cisplatin should also be considered, as they can contribute to the development of hyponatremia [1,24-25].

Abu Zeinah et al. concluded that the in-hospital mortality was related to the severity of hyponatremia. The mortality rate was 4.28 times higher in patients with moderate-to-severe hyponatremia compared to that of normonatremic patients (p ≤ 0.05) [26].

Another cross-sectional study, which included 116 patients with lung cancer, showed that serum sodium levels can influence the ECOG performance status and prognosis. Additionally, hyponatremia was associated with longer hospital stays and higher mortality [24].

A retrospective study of 453 patients with SCLC by Hansen et al. concluded that hyponatremia was associated with lower overall survival rates. Serum sodium levels must be corrected to initiate chemotherapy and ensure favorable outcomes, and this can be achieved via standard fluid restriction, normal or 3% saline, or the use of ADH receptor antagonists such as tolvaptan. Failure to normalize sodium levels within two cycles of chemotherapy was associated with a poorer prognosis (p = 0.027) [27].

The incidence of SIADH in non-small cell lung carcinoma (NSCLC) is extremely rare. A large case series by Sørensen et al. stated that 3 out of 427 patients with NSCLC had SIADH. Similar to SCLC, hyponatremia is an important prognostic factor that could impact clinical outcomes. Hence, early detection and prompt correction of hyponatremia could improve survival rates. When making a treatment plan, physicians should consider blood volume, serum sodium level, neurological symptoms, and social and psychological factors [28,29].

SIADH in pulmonary embolism

Pulmonary embolism (PE) is a major health problem associated with a 30-day mortality of 10%. Among patients who present with PE, hyponatremia is common. Although not a commonly used severity indicator, it contributes to increasing the risk of short-term mortality compared to those without hyponatremia stratified by the pulmonary embolism severity index (PESI) (p = 0.05) [30,31].

A study performed by Tamizifar et al. demonstrated that patients with hyponatremia had a high PESI and mortality up to three times compared to normonatremic patients with PE. This shows that serum sodium levels could be used as a prognostic factor in patients with PE [31].

Hyponatremia influences the neurohormonal activation and the non-osmotic release of vasopressin due to the activation of the sympathetic and the renin-angiotensin-aldosterone axis, which can be seen in pulmonary arterial hypertension with a degree proportional to that of right ventricular dysfunction.

A study conducted by Scherz et al. showed that patients with PE who were hyponatremic on presentation had a higher short-term mortality and an increased risk of hospital readmission based on serum sodium levels. A serum sodium of less than 130 mmol/L had a mortality rate of 28.5% and a readmission rate of 19.3%, whereas a serum sodium of 130-135 mmol/L had a mortality rate of 13.6% and a readmission rate of 15.6%. Normonatremic patients with PE had a mortality rate of 8% and a readmission rate of 11.8% (p ≤ 0.001). However, further research is needed to determine whether the correction of hyponatremia could improve the prognosis for patients with PE, as well as to evaluate whether serum sodium levels can serve as indicators of disease severity and prognosis in these patients [30].

SIADH in interstitial lung disease

The direct association between SIADH and ILD is not well established in medical literature. However, numerous case reports indicated a potential connection between them. A case report from Japan described a patient with acute interstitial pneumonia superimposed on pneumoconiosis who was diagnosed with SIADH during his clinical course. The SIADH resolution corresponded to improving ILD after corticosteroid treatment [32].

Other case reports from Japan described the resolution of SIADH with the treatment of acute interstitial pneumonia. Furthermore, cases from Japan and Korea also showed that ILD can be caused by SIADH-associated drugs such as carbamazepine and cyclophosphamide [33,34].

Raising awareness about the potential link between SIADH and ILD could aid in the early detection of hyponatremia and improve patient outcomes.

Management and treatment of SIADH in respiratory disorders

Acute Management

The management of SIADH involves identifying and treating the underlying etiology, as well as correcting and maintaining serum sodium at normal levels. There are numerous treatment options, and the choice of treatment depends on the severity of symptoms of hyponatremia. A mild but acute hyponatremia can present with severe symptoms such as delirium and seizures, whereas a chronic but severe hyponatremia could potentially be asymptomatic or present with mild symptoms.

Management of Mild-to-Moderate Symptoms

The treatment of hyponatremia with mild-to-moderate symptoms includes fluid restriction, with a goal of less than 1,000 mL of water per day. If this method fails to correct hyponatremia, second-line therapies may include oral salt tablets, tolvaptans, or urea [35].

Oral salt tablets or IV normal saline can be administered. In addition, in patients with a significantly high urine osmolality (more than 500 mOsm/kg), loop diuretics such as furosemide 10-40 mg oral or IV can be added with oral salt tablets to decrease urine concentration and increase water excretion [35,36].

Another treatment option to correct hyponatremia caused by SIADH is vasopressin receptor antagonists such as oral tolvaptan or IV conivaptan. The mechanism by which these drugs work is competitively blocking V2 receptors in renal collecting ducts and preventing ADH-mediated water retention, leading to the correction of hyponatremia. SALT-1 and SLAT-2 trials evaluated the efficacy of tolvaptan in hypervolemic and euvolemic hyponatremia, which showed that diuresis induces the improvement of symptoms or survival, and despite its efficacy in correcting hyponatremia, the risk of rapid correction of serum sodium levels and financial cost limit its widespread use in clinical practice [35,37].

Urea can be used to treat SIADH. It works by increasing free water excretion via an osmotic diuretic mechanism. The dose of urea and urine osmolality determine the amount of free water excretion and the effectiveness of treatment. The dose recommended by the European guidelines to treat SIADH is 0.25-0.50 g/kg/day. The only drawback of urea is the taste, as not all patients can tolerate it [38].

Management of Severe Symptoms

Patients presenting with severe symptoms of hyponatremia, such as delirium, seizures, or coma, should be treated urgently with 3% hypertonic saline, which can also be used in resistant hyponatremia. In these cases, a 100 mL bolus of 3% hypertonic saline should be given in the first 3-4 hours, and sodium levels should be monitored closely and measured within 2-3 hours to adjust further doses. Hypertonic saline is extremely effective in increasing serum sodium levels as its osmolality (1,026 mOsm/kg) is higher than urine osmolality. The use of hypertonic saline beyond the resolution of acute severe symptoms is not recommended as it carries the risk of overcorrection [35].

The rate of correction is an important factor in managing hyponatremia. The correction rate should not exceed 8 mEq/L per day, as rapid correction can lead to osmotic demyelination syndrome (ODS), which can cause impairment to the protective myelin sheath due to osmotic stress and lead to severe CNS complications [35].

Other Treatments

Demeclocycline is uncommonly used to treat hyponatremia caused by SIADH. Its mechanism is not fully understood, but it involves the induction of nephrogenic diabetes insipidus and can cause renal dysfunction as an adverse effect. Since demeclocycline causes polyuria, it can lead to hypernatremia in some cases, especially with fluid restriction. In addition, the dose adjustment can be complicated by the unpredictability of the onset of its action, which can take two to five days. Overall, the safety profile of demeclocycline is variable, with complications such as renal toxicity and hypernatremia. Hence, the European guidelines recommend against the use of this medication [39].

Chronic SIADH management

Awareness of the chronicity of low plasma sodium concentration is the key issue determining the urgency of treatment and the speed of reversal of hyponatremia. Chronic hyponatremia is defined as low sodium levels for at least 48 hours. Due to cellular adaptation, which protects against the development of raised intracranial pressure, patients with chronic hyponatremia are less likely to develop neurological symptoms. However, patients with chronic hyponatremia are vulnerable to ODS caused by aggressive overtreatment [40].

For patients with chronic hyponatremia, correction parameters and therapeutic limits include raising serum sodium levels by 4-6 mEq/L to reverse brain edema and not exceeding 6-8 mEq/L for any 24-hour period to minimize the risk of ODS [41].

Monitoring Sodium Levels

Many experts recommend measuring serum sodium levels at least every 4-6 hours and urine volume hourly in symptomatic patients until hyponatremia reaches 125 mEq/L or higher [41]. Frequent monitoring (every 1-2 hours) is considered in patients who are at risk of complications from rapid correction, i.e., ODS [42].

In asymptomatic patients or those with mild hyponatremia who do not require admission, the frequency of monitoring serum sodium levels depends on clinical judgment, considering the underlying etiology and the nature of the treatment.

Non-pharmacological Therapeutic Treatment

In patients with non-hypovolemic hyponatremia, the first-line management is fluid restriction, with the aim of 500 mL/day below the 24-hour urine volume [41]. An exception to this is patients with subarachnoid hemorrhage because fluid restriction may lead to cerebral vasoconstriction [43].

Pharmacological Therapeutic Treatment

If fluid restriction fails to maintain serum sodium levels above 130 mE/L in patients with SIADH, the second-line treatment options are oral solute or loop diuretics. Examples of oral solutes are sodium chloride salt tablets (1 gram of salt constitutes an osmolar load of 106 mOsm) and urea powder (15 grams of urea constitute an osmolar load of approximately 250 mOsm) [44].

Loop diuretics decrease sodium chloride absorption in the thick ascending loop of Henle, reducing the urine osmolality to 250 to 300 mOsm/kg. Therefore, loop diuretics are effective only when the urine osmolality is higher than the plasma osmolality, usually in ranges above 500 mOsm/kg. Loop diuretics are used in concomitant with oral solutes [45].

The third-line treatment options include drugs such as demeclocycline and antidiuretic (vasopressin) hormone antagonists. Demeclocycline, a tetracycline derivative, induces nephrogenic diabetic insipidus by resisting the action of antidiuretic (vasopressin) hormone. However, it is rarely used in clinical practice due to nephrotoxicity [46].

Antidiuretic (vasopressin) hormone receptor antagonists block the effect of ADH on the V2R receptor, leading to pure water excretion without sodium or potassium excretion. Their clinical utility is limited due to a lack of available data owing to favorable clinical outcomes.

Long-term consequences of chronic hyponatremia and its effects on mortality and cognitive decline

Cognitive Impairment

Cognitive impairment is a symptom commonly associated with hyponatremia, particularly in cases of chronic hyponatremia. Several studies have found an association between chronic hyponatremia and cognitive impairment, even when sodium levels are only mildly or moderately low.

Chronic hyponatremia in older adults is associated with an increased risk of falls, delirium, and cognitive impairment. Even mild hyponatremia can lead to a decline in functional status and independence. Therefore, proper monitoring and management are essential to prevent long-term complications and hospitalization.

In a study conducted by Vaghasiya et al., the performance of patients with hyponatremia on the Mini-Mental State Examination (MMSE) was evaluated, with assessments repeated after their sodium levels were corrected. The results showed an increase in MMSE scores in 93% of patients following improvement in serum sodium concentration (p = 0.001), suggesting that the correction of hyponatremia was the factor driving the improved cognitive performance [47].

In another study conducted by Gunathilake et al., the Audio Recorded Cognitive Screening Tool was used to compare the cognitive performance of participants with hyponatremic to a control group. The results showed that the scores of participants with mild hyponatremia were, on average, 4.67 points lower than those of the control group (95% CI 1.56-7.79; p = 0.01). Significant reductions in cognitive performance were observed even when sodium levels mildly decreased by 5 mmol/L [48].

In a case-control study examining the incidence of falls, 122 patients with asymptomatic mild chronic hyponatremia were compared to 244 controls. The study found that 26 patients (21.3%) with hyponatremia were admitted due to falls compared to just 5.3% of the control group (adjusted odds ratio: 67; 95% CI: 7.5-607; p < 0.001). The frequency of falls was similar regardless of the severity of hyponatremia.

Gait analysis revealed that the total distance traveled by the center of pressure was significantly greater in patients with hyponatremia (1,336 ± 320 mm) compared to those with normal sodium levels (1,047 ± 172 mm; p = 0.003). Attention tests showed that the mean response time was longer in patients with hyponatremia (673 ± 182 milliseconds) compared to controls (615 ± 184 milliseconds), with a difference of 58 milliseconds (p < 0.001).

The study's findings suggest that mild chronic hyponatremia is associated with a high incidence of falls, likely due to significant impairments in gait and attention. Treating these patients may help prevent many hospitalizations [49].

Chronic Hyponatremia Effects on Mortality

There is an overall increase in both mortality and morbidity in ambulatory and hospitalized patients with hyponatremia. Patients with hyponatremia were over seven times more likely to die during hospitalization and more than twice as likely to die after discharge compared to the control group (p < 0.0001 for both) [50]. Additionally, developing hyponatremia during hospitalization further increases the risk of mortality compared to patients who are hyponatremic at admission [51].

Hyponatremia has been associated with higher mortality in patients with heart failure, liver cirrhosis, myocardial infarction, pulmonary hypertension, and PE. A common factor among these conditions is their ability to trigger a neurohumoral response, which aims to restore arterial filling by activating the renin-angiotensin system and increasing vasopressin secretion. This response leads to electrolyte-free water reabsorption by the kidneys, thereby predisposing patients to hyponatremia. In this context, hyponatremia is a marker of the degree of neuro-humoral activation and, by extension, the severity of the underlying disease [52].

Prognosis of patients with SIADH in lung disorders

Impact of SIADH on Disease Progression in COPD

In a prospective observational study conducted in Spain from October 1, 2016, to October 1, 2018, researchers investigated the impact of hyponatremia on the prognosis of COPD exacerbations. A total of 602 patients were enrolled, with a mean age of 73.8 years, and 86% of participants were male. Hyponatremia was recorded in 65 (10.8%) cases. Among the patients, 362 (60%) showed poor outcomes: 18 (3%) died upon admission, 327 (54.3%) had a prolonged hospital stay, and 91 (15.1%) were readmitted within one month of discharge. The most common cause of death was respiratory failure (53%). Patients with worse outcomes were more likely to have been classified as GOLD severe or very severe (p < 0.0001), had worse baseline dyspnea (p < 0.0001), had a higher percentage of flu vaccinations, and had a greater frequency of atrial fibrillation (AF) (p = 0.002), congestive heart failure (CHF) (p = 0.001), and chronic kidney disease (p = 0.002). The study found that factors associated with poor outcomes included hyponatremia, pneumonia, and not using home oxygen. Patients with hyponatremia had a higher prevalence of AF, pleural effusion on chest X-ray, and prolonged hospital stays, with these associations remaining independent of other variables. However, whether hyponatremia has a direct effect on mortality or whether it is a severity marker in certain patients is unknown [53].

The prevalence of hyponatremia is higher in frail patient groups, especially among elderly individuals, where it is observed in nearly half of acute geriatric admissions [54]. Hyponatremia in elderly patients is predominantly mild and chronic (i.e., serum sodium 130-134 mmol/L developing over >48 h). These cases lack the obvious neurological symptoms seen in acute severe hyponatremia due to the homeostatic compensatory mechanisms present in chronic hyponatremia, which allow brain cells to adapt to changes in plasma osmolality over time. As a result, mild chronic hyponatremia is typically considered asymptomatic despite being associated with significant geriatric presentations such as falls and confusion [55].

Discussion

Hyponatremia is a common pathophysiological condition found both in the general population and among hospitalized patients. Increasing evidence indicates that serum sodium disorders, especially hyponatremia, are linked to poor outcomes, including higher mortality, longer hospital stays, and a greater likelihood of readmission within 30 days for patients with various conditions including COPD [56]. Pulmonary diseases, especially pneumonia (whether viral, bacterial, or tuberculous), can result in SIADH, although the exact mechanism remains unclear. A similar response may occasionally occur with conditions such as asthma, atelectasis, acute respiratory failure, and pneumothorax [12].

The pathophysiology of SIADH in chronic conditions such as pulmonary malignancies, especially in tumors of small cell lung cancer, is related to the tumor production of ADH, also known as arginine-vasopressin (AVP). Typically, AVP is a product of the neurohypophyseal system, with the cell bodies of the supraoptic and paraventricular nuclei synthesizing the octapeptides AVP and oxytocin in association with specific carrier proteins, the neurophysins [30]. However, unlike the well-understood mechanism of how SIADH occurs in chronic pulmonary conditions, it is still unclear how SIADH and hyponatremia are associated with acute pulmonary conditions such as asthma and COPD.

Several pathophysiological scenarios have been discussed for SIADH in these diseases, including a reset osmostat, an effect on baroreceptors, or a direct effect of hypercapnia on vasopressin release. Since many of these pulmonary diseases are also characterized by infection, the relation between the acute phase response and hyponatremia, which is becoming increasingly clear, is also of interest. Recent animal data showed an intriguing interaction between interleukin-6 and vasopressin release. Diagnosis can be particularly difficult due to SIADH's nonspecific symptoms, which often resemble those of chronic respiratory diseases. Differentiating SIADH from other causes of hyponatremia in patients with lung diseases requires careful monitoring of electrolyte and fluid balance and exclusion of adrenal or thyroid dysfunctions. This review highlights clinicians' diagnostic difficulties in ruling out other causes of hyponatremia in chronic respiratory cases [57]. SIADH is a common condition with a wide variety of causative diseases. Hyponatremia is associated with high morbidity and mortality. With little prospective randomized controlled trial evidence, much of the guidance and recommendation available for management is based on clinical judgment rather than complex data [4].

In patients with SIADH, the management depends on controlling the underlying etiology and the severity of symptoms. In most cases of mild-to-moderate hyponatremia, fluid restriction is the first-line therapy currently recommended by the European and U.S. hyponatremia guidelines, although evidence for efficacy is limited. For fluid restriction to be effective, there must be a negative fluid balance, with electrolyte-free water output exceeding input. Oral vasopressin V2 receptor antagonist (tolvaptan) blocks AVP action in the kidney, inducing a water diuresis to raise plasma sodium concentration, and is considered a second-line therapy following fluid restriction. The efficacy of tolvaptan versus placebo in raising serum sodium was established in the SALT-1 and SALT-2 trials. Both trials demonstrated greater improvement in their primary end point of serum sodium area under the curve (AUC) with tolvaptan by day 4 (∼4 mmol/L) than with placebo (∼1 mmol/L).

Furthermore, despite the absence of randomized trials, urea is recommended as a second-line treatment after fluid restriction according to the European hyponatremia guidelines and the U.S. expert panel recommendations. Other modalities in managing SIADH and hyponatremia include SGLT2, oral sodium chloride, loop diuretics, demeclocycline, lithium, and apelin analogs. However, those are not first-line therapies and are only last resort. However, severe hyponatremia-causing neurological manifestations such as delirium, seizures, or coma should be treated urgently with 3% hypertonic saline, which can also be used in resistant hyponatremia [36,37,39].

In a prospective study, patients with hyponatremia more frequently reported a history of falling compared with people with normal serum sodium levels (23.8% vs 16.4%, respectively; p < 0.01) and had a higher rate of new fractures over a mean follow-up of 7.4 years (23.3% vs 17.3%; p < 0.004). Hyponatremia is a secondary cause of osteoporosis, falls, and fractures. Mild asymptomatic hyponatremia is linked to an increased risk of bone fractures in ambulatory elderly individuals. Preventing iatrogenic hyponatremia or addressing existing hyponatremia could help reduce the incidence of bone fractures in this population [58-61]. In general, hyponatremia is associated with higher mortality and morbidity, especially in patients with other comorbidities, including respiratory diseases [62-69]. Longer hospitalization was also reported to be associated with hyponatremia [37,70].

Further research is crucial to bridge the existing knowledge gaps regarding SIADH in the context of pulmonary conditions. Rigorous clinical trials targeting SIADH-associated pulmonary disorders are imperative to elucidate effective therapeutic approaches and optimize patient outcomes. Moreover, a comprehensive examination of SIADH's long-term prognosis and recurrence patterns in chronic lung diseases is vital to refine management protocols. Additionally, identifying and characterizing alternative pulmonary triggers of SIADH could unveil novel pathophysiological mechanisms, advancing diagnostic precision and treatment paradigms for this multifaceted condition.

## Conclusions

This review explains the complex relationship between SIADH and chronic respiratory diseases, focusing on its underlying mechanisms, diagnostic challenges, and management difficulties. SIADH not only increases the morbidity and mortality associated with respiratory conditions but also poses unique diagnostic challenges that can complicate patient care. Clinicians must maintain a high level of clinical awareness and diagnostic vigilance, as early recognition of SIADH may significantly influence treatment outcomes. Effective strategies often hinge on a tailored approach that considers the individual patient's profile, including underlying health conditions, the severity of respiratory disease, and the overall clinical picture. While common, interventions such as fluid restriction and pharmacological treatments require careful implementation to avoid potential complications or further deterioration of the patient's condition. Future research should focus on developing targeted therapies and exploring long-term outcomes to improve care standards. By integrating the insights from this review, clinicians can better manage the complexities of SIADH in respiratory diseases, ultimately improving patient outcomes.
